# Cytomegalovirus Colitis in Immunocompetent Patients

**DOI:** 10.7759/cureus.869

**Published:** 2016-11-08

**Authors:** Faisal Inayat, Qulsoom Hussain, Khurram Shafique, Syed H Tasleem, Abu Hurairah

**Affiliations:** 1 Department of Medicine, New York-Presbyterian Hospital, Weill Cornell Medical College, New York City, NY, USA; 2 Department of Medicine, Shifa International Hospital, Shifa College of Medicine, Islamabad, Pakistan; 3 Department of Pathology, SUNY Downstate Medical Center, Brooklyn, NY, USA; 4 Department of Hepatology and Multiorgan Transplant, Beaumont Hospital, Royal Oak, MI, USA; 5 Division of Gastroenterology, Department of Medicine, SUNY Downstate Medical Center, Brooklyn, NY, USA

**Keywords:** colitis, cytomegalovirus, immunocompetent, diagnosis, management, colonoscopy, histopathology

## Abstract

Cytomegalovirus colitis is common in immunocompromised patients, but rare in immunocompetent patients. The present study not only represents the colonoscopy and pathological findings, but also applies the method of diagnosing and treating cytomegalovirus colitis in immunocompetent patients.

## Introduction

Cytomegalovirus (CMV) infections are generally thought to be opportunistic in patients with immunosuppressive diseases like acquired immunodeficiency syndrome (AIDS), underlying malignancies, and organ- or bone marrow-transplantation and patients under treatment with steroids or chemotherapeutics like cisplatin, fluorouracil, leucovorin, epirubicin, vincristine, etoposide, or gefitinib [1].

Recently, a rapidly rising number of literature cases worldwide indicate that CMV infections can also be observed in immunocompetent individuals. It usually results in gastroenteritis, duodenitis, ileitis, proctitis, or exacerbation of inflammatory bowel disease (IBD) [2]. However, colitis secondary to CMV infection is a rare clinicopathologic entity [3-5].

The first report highlighting CMV colitis in an immunocompetent patient was published in 1992 [3]. Since then, several cases have been reported in patients with chronic renal insufficiency and hemodialysis, coinfection with bacterial gastrointestinal infections, and food allergy [1-2]. 

While its association with Crohn's disease is rare, a notable connection between CMV colitis and ulcerative colitis stands out in the literature [1-4]. Because of the overlap in the clinical presentation, accurate diagnosis requires rigorous diagnostic procedures. Furthermore, the diagnositic confusion may also result in implementation of multiple unsuccessful treatment strategies, especially in immunocompetent individuals.

### Materials and methods

In the present study, the biopsy specimens taken from the colonic mucosa were fixed in phosphate buffered formalin at 1:10 dilution for six hours and then processed in a vacuum infiltration processor for 10 hours during which it was treated with buffered formalin (10%), alcohol (80%), alcohol (95%), alcohol (100%), xylene (100%), and then paraffin. It was then manually embedded in paraffin wax, sectioned on a microtome at 5 micrometer thickness and attached to glass slides. For hematoxylin and eosin (H&E) staining, glass slides are stained with routine hematoxylin and eosin staining and submitted for light microscopy.

For CMV staining, 4-5 micrometer sections attached to glass slides was deparaffinized by EZ Prep (Ventana Medical Systems, Inc. Tucson, AZ), then alternately rinsed with reaction buffer and treated with peroxide inhibitor, mouse monoclonal 8B1.2 antibody, linking antibody, horseradish peroxidase (HRP) multimer, OptiView (OV) diaminobenzidine and copper color enhancer. During this process, the slides were incubated for variable period of times at each step before being submitted for examination.

## Case presentation

A 60-year-old male presented to our institution with a 10-year history of intermittent watery diarrhea. He reported 10–12 episodes of loose stools daily. The patient denied frequent travel or chronic medication usage, including antibiotics and non-steroidal anti-inflammatory drugs. His past medical history was significant for myasthenia gravis (MG), chronic kidney disease, iron deficiency anemia, and depression, but no history of IBD. He underwent an uneventful partial colectomy with anastomosis following an appendicular rupture 25 years back.

A general examination revealed a malnourished man with a body mass index of 19.1 (normal, 18.5–24.9). His vitals included a body temperature of 36.5 °C, a heart rate of 81 beats/min and blood pressure of 110/80 mmHg. An abdominal examination revealed a soft, non-tender, non-distended abdomen with normal active bowel sounds. Rebound tenderness and guarding were absent. The rest of the physical examination was unremarkable.

The laboratory results for white blood cells, hemoglobin, hematocrit, platelets, serum creatinine, total protein, albumin, aspartate aminotransferase, alanine aminotransferase, and alkaline phosphatase were within normal limits. The laboratory evaluation was significant for C-reactive protein 18.3 mg/dL (normal <1.0 mg/L), lactate dehydrogenase 351 IU/L (normal 140–280 IU/L), creatinine phosphokinase 237 IU/L (normal 22–198 IU/L), and fasting blood sugar 107 mg/dL (normal 70–100 mg/dL). The coagulation profile was normal. Human immunodeficiency virus (HIV) serology and hepatitis panel were negative.

During the initial days of his hospitalization, the diarrhea worsened and was accompanied by blood in stools. The patient developed a mild fever of 38℃. Electrolyte disturbances (hypokalemia and hypomagnesemia) were attributed to diarrhea and were promptly corrected. The stool cultures were negative, and *Clostridium difficile* toxin was not detected in stool samples.

**Figure 1:**
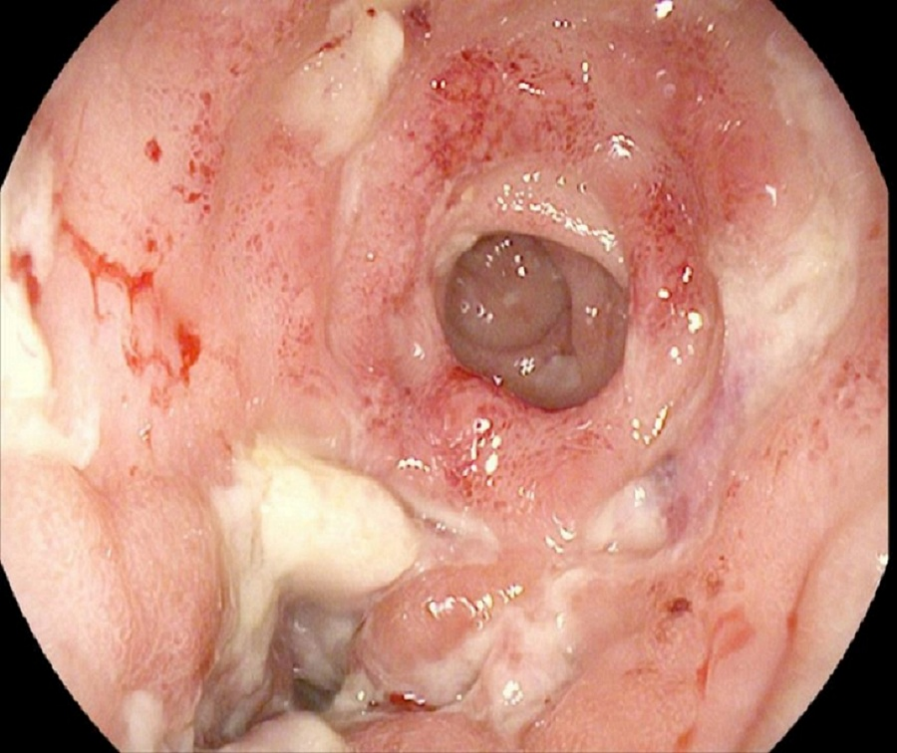
Colonoscopy Diffusely erythematous, friable and ulcerated mucosa was visualized at the anastomotic site involving cecum at 80 cm from the anal verge.

A colonoscopy revealed diffusely erythematous, friable and ulcerated mucosa with few small scattered diverticuli throughout the colon (Figure 1).

**Figure 2:**
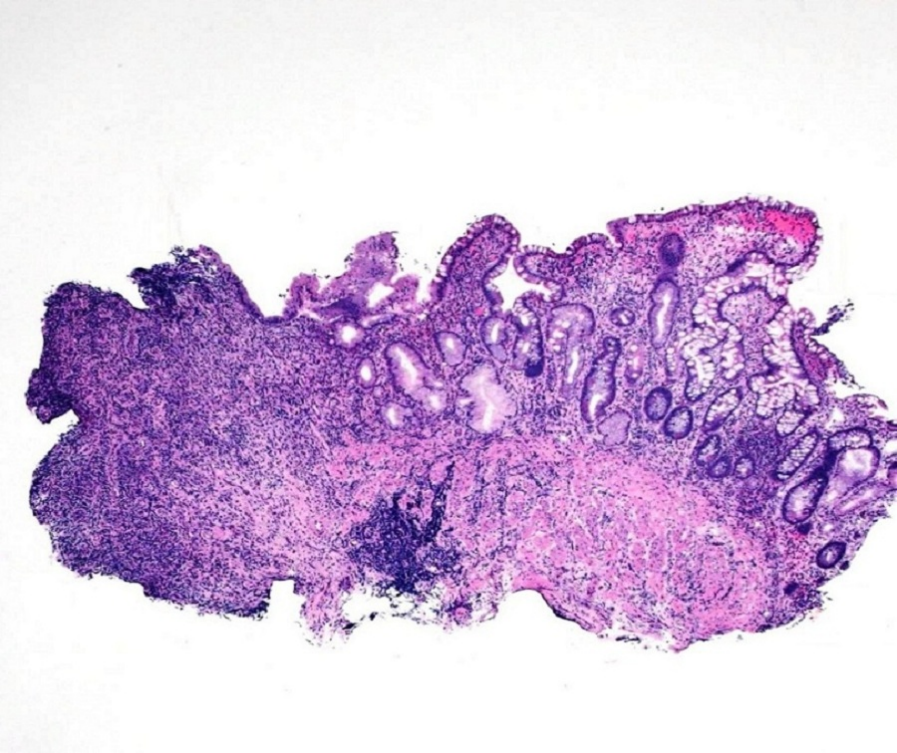
Colon Biopsy Colonic mucosa with adjacent ulceration, acute and chronic inflammation and granulation tissue formation. (H&E, Magnification x20).

The colonic biopsy specimens showed acute and chronic inflammation with granulation tissue formation (Figure 2).

**Figure 3:**
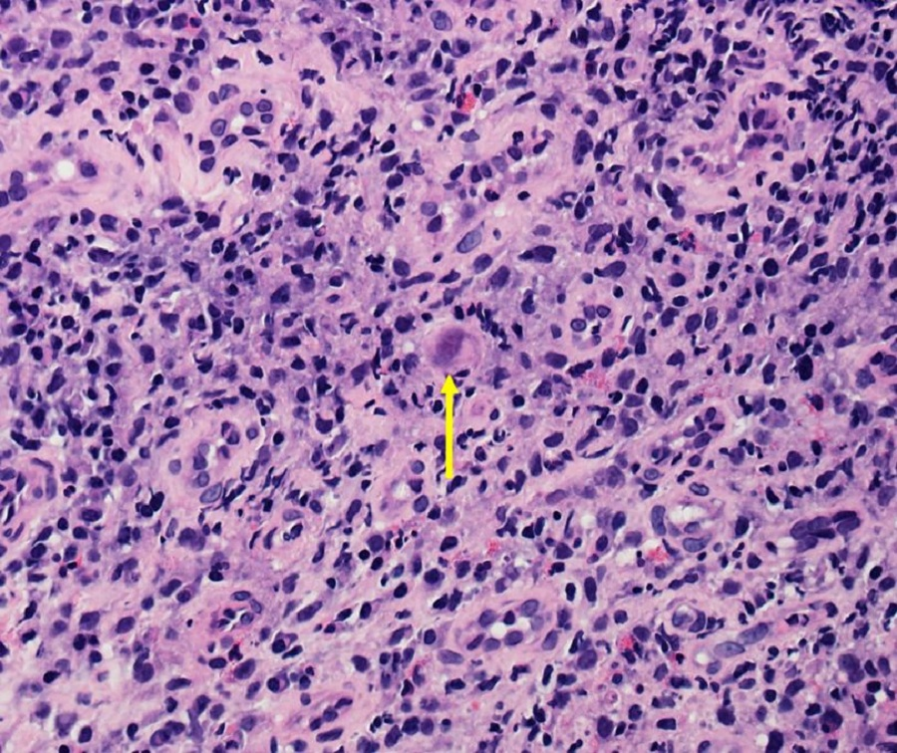
Cytomegalovirus Intranuclear Inclusion Cell infected with cytomegalovirus with smudgy intranuclear inclusion surrounded by granulation tissue. (H&E, Magnification x400).

In a detailed histopathologic examination, a few cells were found to be infected with CMV with evidence of smudgy intranuclear inclusions surrounded by granulation tissue (Figure 3).

**Figure 4:**
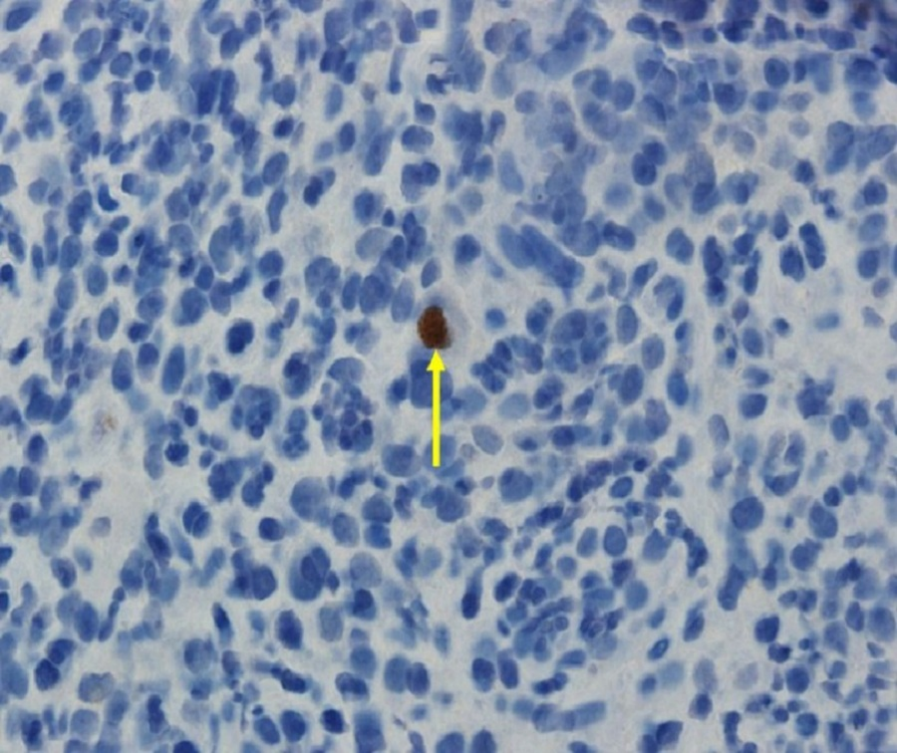
Immunohistochemistry Immunohistochemical analysis showing positive nuclear staining for cytomegalovirus in an infected cell. (CMV Immunostain, Magnification x400).

On immunohistochemical studies, the infected cells demonstrated positive nuclear staining for CMV (Figure 4).

**Figure 5:**
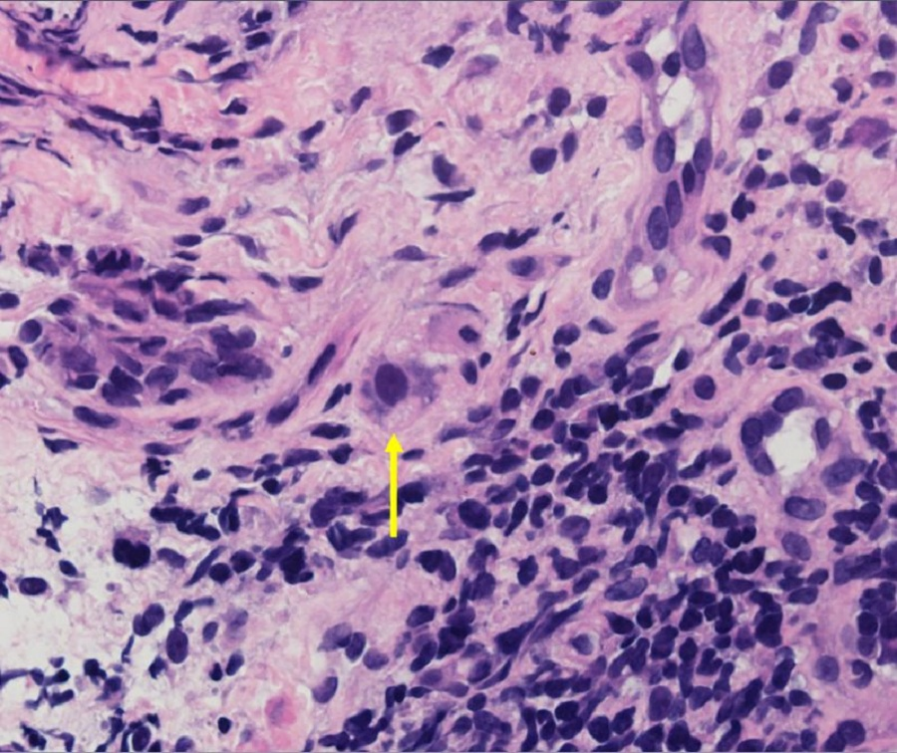
Cytomegalovirus Intranuclear Inclusion Another cell infected with cytomegalovirus showing characteristic cytoplasmic inclusions. (H&E, Magnification x400).

Numerous CMV infected cells were found with cytoplasmic inclusions (Figure 5).

**Figure 6:**
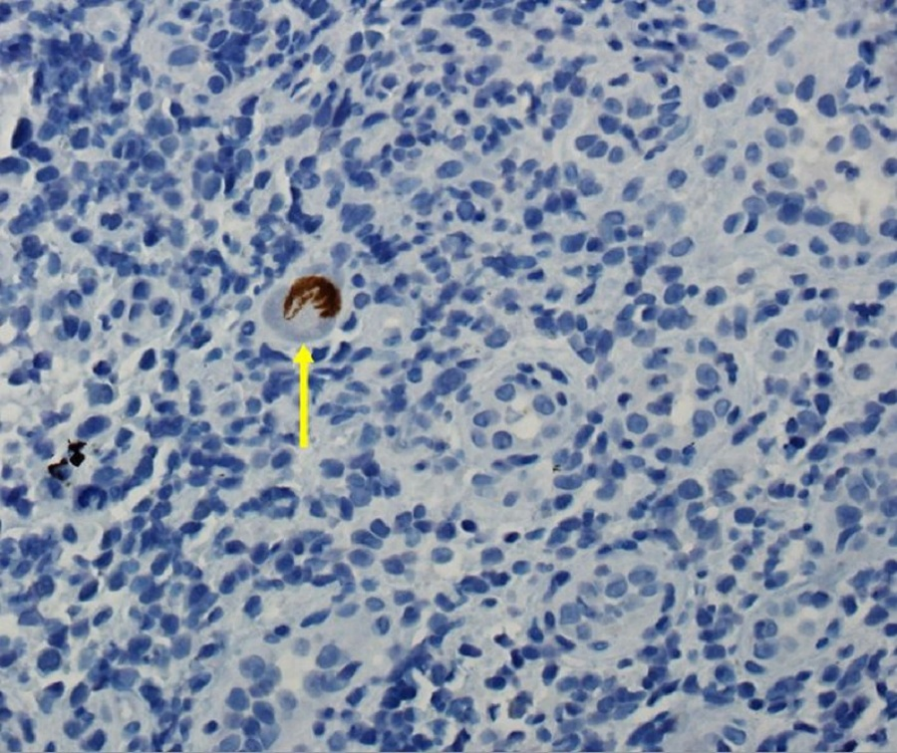
Immunohistochemistry Positive cytomegalovirus immunostain on another infected cell (CMV Immunostain, Magnification x400).

CMV immunostain was positive in these cells (Figure 6).

Furthermore, active CMV infection was confirmed by high immunoglobulin M (IgM) CMV titer and CMV antigen in peripheral blood samples.

After the diagnosis of CMV colitis, the patient was treated with intravenous gancicolivir 200 mg/12 hours as induction therapy for a week followed by valganciclovir for three months. His diarrhea episodes decreased to five to six bowel movements per day within a few days of initial treatment. A follow-up colonoscopy showed disappearance of the colonic ulcers. The patient reported bowel movements to be more formed and he started gaining weight appropriately.

## Discussion

CMV is a double-stranded DNA virus belonging to the herpes virus family. The prevalence of CMV infections in the general adult population is approximately 40%–100% [1]. CMV infections are common worldwide due to its excretion in body fluids and transmission by close personal contact [2]. In normal hosts, primary infection is usually asymptomatic, but sometimes can result in mononucleosis-like syndrome, accompanied by symptoms such as fever, myalgia, cervical lymphadenopathy and elevated liver enzymes [2].

Several studies have shown that CMV can cause severe disease in immunocompromised patients; for instance, patients who have been treated with steroids, have renal failure, AIDS, cancer or IBD, or have undergone bone marrow or solid organ transplants [3-10]. Galiatsatos, et al. performed a meta-analysis of CMV colitis among immunocompetent patients and included 44 cases over 23 years [5].

CMV can infect the entire gastrointestinal tract. However, the colon is the most frequent site in immunocompetent patients [5]. CMV colitis clinically presents with a low-grade fever, weight loss, anorexia, malaise, and abdominal pain [6]. Furthermore, watery diarrhea and hematochezia are other common manifestations. Mucosal hemorrhage and perforation can be designated as life-threatening complications of CMV colitis [6].

Colonoscopy can reveal a range of CMV colitis-related findings. Most commonly identified endoscopic abnormalities were well-demarcated ulcerations (50%), ulceroinfiltrative changes (25%), and pseudomembrane formation (25%) [7]. Biopsies are essential because similar findings can be present in other types of colitis. A histopathologic analysis typically reveals diffuse ulcerations and necrosis with scattered large 25 to 35 µm cells containing basophilic intranuclear CMV inclusions that play an active role in colonic mucosal damage [8].

Diagnosis of CMV colitis requires both serologic and histologic findings [6]. Immunohistochemistry of the biopsied tissue using monoclonal antibodies and in situ DNA hybridization enhances the sensitivity of the histopathologic analysis [8]. Positive IgM titer for CMV, CMV antigen in the blood and positive polymerase chain reaction (PCR) in blood or urine are employed to confirm the diagnosis. These characteristics correlated with the histopathologic findings in our patient.

The exact mechanism of the pathogenesis of CMV colitis remains to be determined. However, it has been suggested that CMV can proliferate in the endothelial cells in blood vessels, leading to vasculitis and small vessel thrombosis with local ulceration, which may lead to ischemic colitis [9]. Furthermore, pre-existing ischemic colitis or IBD may also help create a favorable environment for CMV by destroying the colonic mucosa and creating local immunosuppression. CMV colitis may occur under such mucosal conditions and can contribute to poor prognosis, especially in patients with acute severe ulcerative colitis [4].

Recently, a potential association between CMV colitis and drugs treating myasthenia gravis, especially mycophenolate mofetil (MMF) has been described [10]. MMF is used for immunosuppression in myasthenia gravis. Chronic MMF therapy in MG patients may trigger life-threatening infections including CMV and Epstein-Barr virus infections, especially in the elderly. However, at the time of diagnosis of CMV colitis, our patient was off MMF therapy.

There are several agents available for the systemic therapy of CMV infection, including ganciclovir, valganciclovir, foscarnet, and cidofovir. The efficacy and toxicities of these agents have been evaluated extensively only in immunocompromised patients. However, clinical utility of these agents in the immunocompetent hosts remains unproven. Doses of the drugs and the duration of therapy also have not been clarified for this group of patients.

Currently, ganciclovir is the preferred antiviral drug for the treatment of CMV infection in immunocompetent patients. The prognosis in patients with CMV colitis can be improved markedly by both ganciclovir and foscarnet [8-10]. However, ganciclovir can result in serious adverse reactions, including myelosuppression, central nervous system disorders, hepatotoxicity and nephrotoxicity [2]. Despite these serious side effects, antiviral therapy is recommended for immunocompetent patients because without antiviral treatment CMV colitis results in poor outcomes [5].

In our patient, CMV colitis was correctly diagnosed with colonoscopy findings followed by positive serology and immunohistochemical staining. Implementation of intravenous ganciclovir therapy led to a significant clinical improvement, considerable healing and disappearance of characteristic colonoscopy findings. No ganciclovir-related side effects were detected.

## Conclusions

CMV colitis should be considered in immunocompetent patients, especially after exclusion of more common etiologies for severe diarrhea. The diagnosis of CMV colitis can be established on the basis of biopsies or PCR tests. Timely diagnosis and treatment are essential in order to improve the outcome in elderly patients or patients with serious comorbidities. Furthermore, population-based clinical studies are warranted to broaden the scope of our knowledge on CMV colitis in immunocompetent patients and to frame guidelines to standardize the care of these patients.

## Appendices

The patient agreed to participate and was explained the nature and objectives of this study, and informed consent was formally obtained. No reference to the patient's identity was made at any stage during data analysis or in the report.
